# NLRP3 Inflammasome Activation in Cancer: A Double-Edged Sword

**DOI:** 10.3389/fimmu.2020.01444

**Published:** 2020-07-08

**Authors:** Shaima'a Hamarsheh, Robert Zeiser

**Affiliations:** ^1^Department of Medicine I, Medical Center—University of Freiburg, Faculty of Medicine, University of Freiburg, Freiburg, Germany; ^2^Faculty of Biology, University of Freiburg, Freiburg, Germany; ^3^German Cancer Consortium (DKTK) Partner Site Freiburg, German Cancer Research Center (DKFZ), Heidelberg, Germany; ^4^Comprehensive Cancer Center Freiburg (CCCF), University of Freiburg, Freiburg, Germany; ^5^Center for Biological Signalling Studies (BIOSS) and Center for Integrative Biological Signalling Studies (CIBSS), University of Freiburg, Freiburg, Germany

**Keywords:** NLRP3, inflammasome, cancer, therapeutic targets, activation

## Abstract

Inflammation is involved in tumor development and progression as well as antitumor response to therapy. In the past decade, the crosstalk between inflammation, immunity, and cancer has been investigated extensively, which led to the identification of several underlying mechanisms and cells involved. The formation of inflammasome complexes leads to the activation of caspase-1, production of interleukin (IL)-1β, and IL-18 and pyroptosis. Multiple studies have shown the involvement of NLRP3 inflammasome in tumorigenesis. Conversely, other reports have indicated a protective role in certain cancers. In this review, we summarize these contradictory roles of NLRP3 inflammasome in cancer, shed the light on oncogenic signaling leading to NLRP3 activation and IL-1β production and outline the current knowledge on therapeutic approaches.

## Introduction

It is well-established that inflammation caused by viral or microbial infections contributes to tumorigenesis. However, emerging evidence have shown that it as well has a pivotal role in most stages of cancer development, besides interfering with the ability of immune system to counteract tumor cells and affecting response to treatment. These mechanisms are mainly driven by innate and adaptive immune cells, such as dendritic cells, macrophages, natural killer (NK) cells, neutrophils, and lymphocytes (1, 2).

One of the central mechanisms contributing to inflammation in immune cells is mediated by special cytoplasmic protein complexes known as inflammasomes. They are divided based on their structural features into nucleotide-binding and oligomerization domain (NOD)-like receptors (NLRs) and absent in melanoma 2 (AIM2)-like receptors (ALRs). In addition, inflammasomes belong to a larger family of receptors known as pattern recognition receptors (PRRs), where their function is the recognition of pathogen- or danger-associated molecular patterns (PAMPs or DAMPs), causing the activation, maturation, and production of pro-inflammatory cytokines (3). Besides, emerging evidence has proposed that inflammasomes act as a “signal integrator” detecting changes in cytoplasmic homeostasis. These perturbations, named as homeostasis-altering molecular processes (HAMPs), are induced by the functional consequences of cellular processes, where the inflammasome responds to a cellular imbalance rather than a molecular pattern, triggering inflammation in a sterile context. This provides hints that inflammasome activation via the HAMP detection pathway might also be involved in disease pathogenesis (4, 5).

Amongst the inflammasomes family, NLRP3 inflammasome is the most characterized. Mutations in NLRP3 are associated with several autoimmune and inflammatory diseases, particularly a group known as cold-induced auto-inflammatory syndrome (CAPS). In addition, NLRP3 has been implicated in several other diseases including inflammatory bowel disease (IBD), rheumatoid arthritis, and Parkinson's disease (6). In cancer, analysis of copy number alterations in tumor samples has shown *NLRP3* with a high frequency of copy gains, thus acting more as an oncogene (7). However, different roles of inflammasomes in tumorigenesis and antitumor immunity have emerged in the past decade (8), without overlooking the well-established role of cytokines in cancer pathogenesis (9).

Here, we discuss the structure and activation pathways of NLRP3, and provide a brief updated review on the most recent research investigating its opposing roles in cancer. Lastly, we list the potential therapeutic targets and the latest reports and clinical trials investigating them.

## NLRP3 Inflammasome

### Historical Background

Since the cloning of IL-1β in 1984 (10, 11) and the characterization of its various immunological activities, enormous research has been conducted to further explore the biology of cytokines and their effects on inflammation and other physiological roles. The first major contribution following this, was the identification of IL-1-converting enzyme (ICE), now named as caspase-1 (12, 13). Despite that, the underlying mechanisms causing the processing and release of IL-1ß remained unclear. It was only until 2002, when Martinon et al. (14) identified a caspase-activating complex, which leads to the maturation and secretion of IL-1β, now known as the inflammasome. They continued their pioneering work in this field (15), which led to discovering the association of inflammasomes with CAPS (16), as well as gout and type 2 diabetes. Additionally, they reported several inflammasome agonists, PAMPs including muramyl dipeptide (MDP) (17), viral DNA (18) and malaria-associated hemozoin (19); DAMPs such as monosodium urate (MSU) crystals (20); and environment-derived factors like asbestos, silica (21) and alum (22). A number of different clinical trials for inflammasome-related inflammatory diseases were conducted which led to the development of a therapy for CAPS patients in the clinic (23), in addition to promising results in several clinical studies involving gouty arthritis patients treated with anakinra (24, 25). These revolutionary discoveries paved a new path in the fields of inflammasome activation, innate immunity cytokines production, and their involvement in health and disease.

### Structure and Activation of the NLRP3 Inflammasome

**Figure 1:**
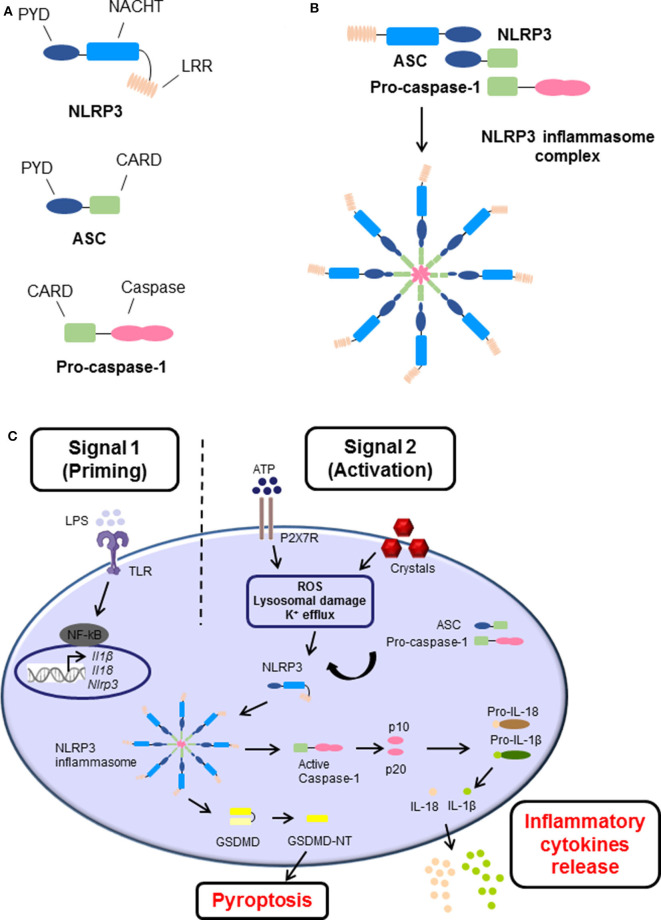
The structure and canonical activation of the NLRP3 inflammasome complex. **(A)** The structure of NLRP3 is comprised of three main domains: (i) NLRP3, containing an N-terminal pyrin domain (PYD), a central NACHT domain, and a C-terminal leucine-rich repeat (LRR) domain; (ii) adaptor apoptosis speck (ASC) which contains PYD and CARD domains; and (iii) pro-caspase-1 which contains caspase-1 and CARD domains. **(B)** Upon activation, NLRP3 undergoes oligomerization, recruits, and binds ASC, which subsequently recruits and binds pro-caspase-1 via their shared domains. The formation of this NLRP3 inflammasome cluster results in a prion-like assembly of the complex. **(C)** The activation process of NLRP3 inflammasome consists of two main signals: (i) Signal 1 (Priming), which is induced by pathogen recognition receptors (PRRs) such as toll-like receptors (TLRs) activated by pathogen-associated molecular patterns (PAMPs) such as lipopolysaccharide (LPS), or other endogenous factors and mechanisms such as reactive oxygen species (ROS), hypoxia, metabolites, oxidized low-density lipoprotein (oxLDL), amyloids, and complement (not shown). This leads to the transcriptional upregulation of *Nlrp3, Ill1b*, and *Il18* via transcription factors such as NF-κB. (ii) Signal 2 (Activation), is provided by PAMPs or damage-associated molecular patterns (DAMPs), such as adenosine triphosphate (ATP) and crystals which activate different signaling events including ROS, lysosomal damage and K^+^ efflux, leading to activation and recruitment of NLRP3, oligomerization, and formation of NLRP3 inflammasome complex. The activation and formation of NLRP3 inflammasome has two main consequences: (i) cleavage of Gasdermin D GSDMD and inducing pyroptosis and/or (ii) auto-cleavage and formation of the active caspase-1 and p10/p20 tetramer which then proteolytically cleaves pro-IL-1β and pro-IL-18 into their bioactive forms IL-1β and IL-18 prior to their release.

Inflammasomes are danger-sensing, multimeric protein complexes that are part of the innate immune response. The most widely studied and well-characterized inflammasome is NLRP3, which is characterized by the presence of a central nucleotide-binding and oligomerization (NACHT) domain, which is usually flanked by C-terminal leucine-rich repeat (LRR), and N-terminal pyrin domain (PYD) (Figure 1A) (3). In brief, a danger signal sensed leads to a conformational change of NLRP3 causing the exposure of NACHT domain. NLRP3 undergoes oligomerization by homotypic interactions between NACHT domains. As a result, the PYD domain of NLRP3 becomes exposed, recruit the adaptor apoptosis speck protein (ASC, also known as PYCARD) and bind through their shared PYD domains (Figure 1A). Following, ASC converts to a prion-like form and generates long ASC filaments. This interaction recruits the CARD of pro-caspase-1 facilitating its binding to the complex. Additionally, the clustering of pro-caspase-1 forms its own prion-like filaments that separates from the ASC filaments allowing the auto-cleavage and formation of the active caspase-1 p10/p20 tetramer, which then processes cytokine pro-forms into active molecules. Therefore, the cluster of oligomerized NLRP3-ASC-pro-caspase-1 complex results in the assembly of the multi-subunit wheel-shaped inflammasome complex (Figure 1B) (3, 14, 26–29). The activation of NLRP3 inflammasome causes two main effects, the induction of programmed cell death known as pyroptosis, and/or a pro-inflammatory response caused by the release of inflammatory cytokines IL-1β and IL-18.

The canonical activation process requires two main steps known as priming signal and activating signal (Figure 1C). The first step is provided by inflammatory stimuli from toll-like receptors (TLR) ligands or endogenous molecules, which induce the expression of NF-κB. Additionally, other endogenous factors and mechanisms have been identified to prime the inflammasome in sterile inflammatory diseases, such as reactive oxygen species (ROS), hypoxia, metabolites, oxidized low-density lipoprotein (oxLDL), amyloids, and complement. The second step is usually promoted by PAMPs and DAMPs, which cause potassium ion (K^+^) efflux, calcium (Ca^+2^) flux, lysosomal damage or ROS production leading to NLRP3 inflammasome assembly, caspase-1 cleavage, and thus the maturation and secretion of IL-1β and IL-18 (27, 28, 30).

On the other hand, other pathways for NLRP3 inflammasome activation were described (reviewed elsewhere (31, 32). The non-canonical NLRP3 inflammasome pathway is activated by most Gram-negative bacteria, and requires capase-11 (33) as well as vacuolar rupture mediated by interferon-inducible guanylate-binding proteins (GBPs). Also, an alternative NLRP3 inflammasome pathway is activated in human monocytes induced by LPS and requires the molecules RIPK1, FAS-associated death domain protein (FADD), and caspase-8 (34).

### NLRP3 Inflammasome in Cancer

The function of NLRP3 inflammasome in human cancers is rather a conflicting topic (8, 35), where there is evidence of a protective anti-tumorigenic effect as well as a pro-tumorigenic role in different types of cancer (summarized in Table 1). Here, we discuss both roles shown in murine and human studies and introduce new insights for the effect of oncogenic mutations in inducing NLRP3 inflammasome activation in leukemias.

**Table 1:** The dual effect of NLRP3 inflammasome in cancers.

**Type of cancer**	**Role and mechanism of action**	**References**
**Pro-tumorigenic role**		
Breast cancer	NLRP3 and IL-1β promoted tumor growth and metastasis via infiltration of myeloid cells (MDSCs and TAMs) providing an inflammatory microenvironment	(36)
	Murine and human cancer-associated fibroblasts sense DAMPs and activate NLRP3 inflammasome pathway leading to IL-1β secretion	(37)
	IL-1β in a TNBC mouse model has an immunosuppressive, pro-tumorigenic role in the TME, and blocking it improves checkpoint inhibition by anti-PD1	(38)
	S1PR1 on TAMs is associated with NLRP3 expression and correlated with lymphangiogenesis and metastasis	(39)
Colon cancer	NLRP3 is highly expressed in mesenchymal-like colon cancer cells (SW620). NLRP3 is upregulated in colon cancer epithelial cells HCT116 and HT29 during EMT via TNF-α and TGF-β1	(40)
Colorectal cancer	NLRP3 polymorphisms are correlated with poorer survival in patients with invasive CRC patients	(41)
	NLRP3 senses tissue damage, promotes IL-18 which downregulates IL-22BP leading to IL-22 production and promoting tumor development at later stages	(42)
Epithelial skin cancer	IL-1 and caspase-1 play a role in tumor development. ASC expressed in infiltrating myeloid cells acts as a driver of tumorigenesis	(43)
Fibrosarcoma	NLRP3 acts as a suppressor of NK cell antimetastatic function and CD11b^+^Gr-1^intermediate^ (Gr-1^int^) myeloid cells causing decreased levels of CCL5 and CXCL9	(44)
Gastric cancer (GC)	NLRP3 inflammasome activation and IL-1β secretion is upregulated in GC, induce epithelial cells proliferation and tumorigenesis by binding to cyclin-D1 promoter which could be reversed by miRNA-22	(45)
HNSCC	P2X7 and NLRP3 is upregulated in carcinoma tissues and had a role in survival and invasiveness of HNSCC	(46)
	NLRP3 is associated with inflammation-induced carcinogenesis and CSCs markers	(47)
	NLRP3 is overexpressed in human HNSCC tissues, and IL-1β levels were increased in their peripheral blood	(48)
Leukemias (CMML, JMML, and AML)	NLRP3/IL-1β cause myeloproliferation and cytopenias in KRAS-mutant leukemias, mediated by RAC1 activation and ROS production	(49)
LSCC	NLRP3 expression is higher in human cancer tissues compared to normal tissues. High expression of NLRP3 and IL-1β is correlated with a poorer prognosis	(50)
Lung cancer	NLRP3 inflammasome activation enhances the proliferation and metastasis of lung adenocarcinoma cell line A549, mediated by AKT, ERK1/2, CREB, and upregulation of SNAIL	(51)
Lymphoma	NLRP3 inflammasome, through IL-18, promotes lymphoma cell proliferation and inhibits apoptosis, via upregulation of C-MYC, BCL2, and downregulation of TP53 and BAX	(52)
Melanoma	Inhibition of NLRP3 by thymoquinone suppresses metastasis of murine and human melanoma cells by deregulation of IL-1β and IL-18	(53)
	NLRP3 is activated in human melanoma cells, but also constitutively secrete IL-1β via NLRP3 and IL-1R in the absence of exogenous stimulation	(54)
Myelodysplastic syndromes (MDS)	NLRP3 inflammasome is overexpressed in MDS HSPCs, drives clonal expansion and pyroptosis via alarmin signals, gene mutations, and ROS production.	(55)
Pancreatic ductal adenocarcinoma	NLRP3 promotes differentiation of CD4^+^ T cells into tumor promoting Th2 cell, Th17, and regulatory T cell population and suppresses cytotoxic CD8^+^ T cell, mediated by IL-10	(56)
Prostate cancer	Hypoxia causes priming of NLRP3 and AIM2 through upregulation of their receptors and pro-IL-1β	(57, 58)
**Anti-tumorigenic role**		
Colitis-associated cancer (CAC)	NLRP3, PYCARD, or caspase-1 deficiency causes worse disease outcome and morbidity via increased IL-1β and IL-18 secretion	(59)
	NLRP3 or ASC and caspase-1 deficiency leads to higher susceptibility to DSS-induced colitis and mortality rate due to decreased IL-18 levels	(60)
Colorectal cancer (CRC)	Lack of NLRP3 or caspase-1 causes reduced tumor burden due to decreased levels of IL-18 and impaired production and activation of IFN-γ and STAT1	(61)
	NLRP3 inhibits CRC metastatic growth in the liver by IL-18, NK cells, and increased expression of FasL	(62)
	NLRP3 senses tissue damage, promotes IL-18 which downregulates IL-22BP leading to IL-22 production and exerting protective effects against intestinal tissue damage at the peak of inflammation	(42)
Hepatocellular carcinoma (HCC)	NLRP3 inflammasome components were absent or significantly downregulated in human HCC. NLRP3 deficiency is correlated with advanced stages	(63)
Melanoma	NLRP3 inflammasome impairs anti-tumor response by facilitating migration of myeloid-derived suppressor cells (MDSCs)	(64)

*AML: acute myeloid leukemia, CMML: chronic myelomonocytic leukemia, CSCs: cancer stem cells, DAMPs: Danger associated molecular patterns, DSS: dextran sodium sulfate, EMT: epithelial-mesenchymal transition, HNSCC: Head and neck squamous cell carcinoma, HSPCs: hematopoietic stem and progenitor cells, IL-22BP: IL-22- binding protein, JMML: juvenile myelomonocytic leukemia, LSCC: laryngeal squamous cell carcinoma, NK: natural killer, PDAC: Pancreatic ductal adenocarcinoma, ROS: reactive oxygen species, S1PR1: S1P receptor 1, TAMs: Tumor associated macrophages, TGF-β1: transforming growth factor-β1, Th2: T helper type 2 cell, TME: tumor microenvironment, TNF-α: tumor necrosis factors-α, Triple Negative Breast Cancer (TNBC)*.

NLRP3 inflammasome have been shown to promote the development of several cancers, where most studies were focused on proliferation, survival, metastasis, angiogenesis, and immunosuppression. In breast cancer, NLRP3 inflammasome, and IL-1β production promote the infiltration of myeloid cells such as myeloid-derived suppressor cells (MDSCs) and tumor-associated macrophages (TAMs), providing an inflammatory microenvironment thus promoting breast cancer progression (36). In addition, NLRP3 inflammasome in fibroblasts is further linked with progression and metastasis (37), and IL-1β was found to have an immunosuppressive, pro-tumorigenic in the tumor microenvironment (38). Besides, the NLRP3 inflammasome seems to be an effector for promoting metastasis via the lymphatic system and favoring mammary carcinoma development (39). Interestingly, Huber et al. (42) demonstrated that IL-18 induced by NLRP3 causes the downregulation of the soluble IL-22 receptor, IL-22-binding protein (IL-22BP), leading to an increase in the ratio of IL-22/IL-22BP, which at later stages promotes tumor development (42). Additionally, NLRP3 deficiency leads to suppression of metastases and methylcholanthrene (MCA)-induced sarcomas in mouse models, which were dependent on NK cell and IFN-γ (44). In epithelial skin cancer, mice deficient for IL-1R and caspase-1 showed partial protection against skin cancer development (43). Besides, the roles of inflammasomes in melanoma pathogenesis is established (65). In particular, NLRP3 inflammasome was shown to be constitutively expressed and activated in human melanoma cells. However, these cells secrete biologically active IL-1β in an autonomous way without the presence of exogenous stimuli at late stages of the disease (54). In HNSCC, NLRP3 inflammasome is found upregulated in carcinoma tissues and associated with carcinogenesis and cancer stem cells (CSCs) self-renewal activation (46–48). Also, NLRP3 signaling seems to drive immunosuppression in pancreatic carcinoma, by promoting tolerogenic T cell differentiation and adaptive immune suppression via IL-10 (56).

In hematological malignancies, the role of NLRP3 inflammasome in normal and malignant hematopoiesis has been lately reviewed (66). We have recently reported a novel function of the NLRP3 inflammasome in the pathogenesis of hematological malignancies, particularly myeloproliferation in leukemias. Interestingly, and despite the manifestation of oncogenic *KRAS* in hematopoietic cells, we could show that the NLRP3 inflammasome has a key role in the development of several myeloid leukemias features *in vivo*, including cytopenias, splenomegaly, and myeloproliferation. These phenotypes are often seen in chronic myelomonocytic leukemia (CMML), juvenile myelomonocytic leukemia (JMML) and more rarely acute myeloid leukemia (AML) patients harboring *KRAS* mutations. Additionally, we found evidence of NLRP3 inflammasome activation upon analyzing JMML, CMML, and AML patient samples harboring *KRAS* mutations, providing a stronger evidence of the participation of NLRP3 inflammasome in the disease development (49). An open question remains how the NLRP3 inflammasome activation drives hematological malignancies, whether by a cell-autonomous signal that promotes cell proliferation directly or via a modification of the TME or both.

Conversely, NLRP3 inflammasome was also shown to have an anti-tumorigenic role. Previously, Ghiringhelli et al. (67) have proposed that NLRP3 inflammasome is required for dendritic cell-mediated priming of IFN-γ-producing T lymphocytes against tumor cells. NLRP3 inflammasome seems to act as a negative modulator of tumorigenesis in colitis-associated cancer (59), which is confirmatory to the study emphasizing the role of NLRP3 inflammasome in the regulation of intestinal homeostasis and thus protection against colitis (60). In addition, NLRP3 inflammasome deficiency seems to cause increased tumor burdens in colorectal cancer. Moreover, Dupaul-Chicoine et al. (62) reported that NLRP3 inflammasome-mediated IL-18 production suppresses colorectal cancer metastatic growth in the liver. In contrast to the tumor-promoting function of IL-22 discussed above, NLRP3/IL-18-mediated downregulation of IL-22BP under controlled production can also provide protective roles against intestinal tissue damage during the inflammation peak (42). In melanoma, it was shown that NLRP3 in the TME weakens the anti-tumor immune response to a cancer vaccine, by assisting the migration of myeloid-derived suppressor cells (MDSCs), thus suppressing the T cell response (64).

NLRP3 inflammasome signaling in humans is controlled by a variety of factors, such as genetic polymorphisms and mutations that can affect gene expression and ultimately lead to its activation. These effects were seen in patients with inflammatory diseases (68–71). Similarly, genetic polymorphisms involved with NLRP3 inflammasome have also been linked to cancer. For instance, a single-nucleotide polymorphism (SNP) in the *NLRP3* gene, *Q705K* (rs35829419), was correlated with poorer survival in patients with invasive colorectal cancer (41), postulated as a risk allele for sporadic metastatic melanoma in Swedish males (72), and also occurs at high frequency in pancreatic cancer patients (73). Additionally, those with NLRP3 polymorphisms (rs10754558 and rs4612666) are more susceptible to gastric cancer when infected with *Helicobacter pylori* (74). In hematological malignancies, polymorphisms restricted only to *IL-1*β and *IL-18* were associated with clinical and pathophysiological characteristics in AML and chronic myeloid leukemia (CML) (75, 76). Besides, studies utilizing gene expression profiling have also implicated the upregulation of NLRP3 inflammasome in several cancers. For example, NLRP3 is overexpressed in HNSCC, LSCC, and squamous cell carcinoma tissues compared to normal tissues, and often correlated with poor prognosis and worse pathology (48, 50, 77). In bladder cancer, high expression of NLRP3 inflammasome is also found, making it a potential biomarker for its diagnosis (78). Further studies will be required to understand the association between genetic polymorphisms or differential expression of NLRP3 inflammasome and clinical features of cancer.

The understanding of this crosstalk between immunity, inflammasomes, inflammation, and cancer is the foundation for implementing anti-inflammatory therapeutic options in cancer prevention and treatment.

## Therapeutic Potential of Targeting NLRP3 Inflammasome in Cancer

The involvement of the NLRP3 inflammasome in several inflammation-related diseases, including cancer, provided it as an attractive potential target in designing new drugs for treatment. Several reported molecules and drugs were shown to regulate the inflammasome activity. However, many indirectly affect the inflammasome effector functions by targeting other molecules. Until today, current treatment of NLRP3 inflammasome-related diseases in the clinic involve targeting IL-1β or IL-1β receptor by monoclonal IL-1β antibodies or recombinant IL-1 receptor antagonists. Nevertheless, several specific small-molecule compounds have been shown to have anti-inflammatory effects. Here, we review the variety of NLRP3 inflammasome inhibitors which either target components of its canonical signaling pathway or are specific to NLRP3 protein (summarized in Table 2).

**Table 2:** A list of compounds targeting NLRP3 inflammasome either indirectly or directly and their therapeutic potential in cancers.

**Compound name**	**Mechanism of action**	**Reference**	**Studies in cancer**
**1. Targets of NLRP3 inflammasome pathway**			
**NLRP3 inflammasome effectors**			
Anakinra	Interleukin-1 receptor inhibitor	(79)	(80–84)
Canakinumab	IL-1β inhibitor	(23, 85)	(86)
**NLRP3 inflammasome activators**			
P2X7 receptor inhibitors	P2X7R inhibitors	(87)	(46, 88)
**NLRP3 inflammasome expression**			
Andrographolide	NF-κB inhibitor	(89, 90)	(91–95)
Parthenolide	NF-κB inhibitor	(96)	(97–99)
**2. Targets of NLRP3 inflammasome components**			
Thalidomide	Caspase-1 inhibitor	(100)	(101–107)
VX-765	Caspase-1 inhibitor	(108)	–
Pralnacasan	Caspase-1 inhibitor	(109)	–
Ac-YVAD-CHO	Caspase-1 inhibitor	(110, 111)	–
**3. Direct targets of NLRP3 protein**			
MCC950	Directly binds to the Walker B motif of NACHT domain, blocking ATP hydrolysis, and formation of NLRP3 inflammasome	(112, 113)	(48, 49, 55)
Oridonin	NACHT domain and Oridonin share cysteine 279 binding site	(114)	(115, 116)
CY-09	Directly binds NLRP3 motif, leading to the abrogation of ATP binding to NLRP3	(117)	–
OLT1177	Binds to NLRP3 inhibiting its ATPase activity	(118, 119)	–
Tranilast	Directly binds to the NACHT domain of NLRP3 and inhibition of ASC oligomerization	(120, 121)	–

Anakinra is a recombinant form of interleukin-1 receptor antagonist (IL-1Ra) (79), which was approved by the US Food and Drug Administration (FDA) for the treatment of rheumatoid arthritis patients and autoinflammatory disorders (122, 123). We have recently reported that treating *Kras*^*G*12*D*^-mutant leukemia mouse models with anakinra improves myeloproliferation and cytopenia phenotypes (49). Due to its clinical safety record and short life, anakinra is an ideal drug to be used in conjugation with chemotherapy. Indeed, one clinical trial on metastatic colorectal cancer reported that the treatment of anakinra besides fluorouracil (5-FU) plus bevacizumab showed survival benefit (80), while another showed improved outcome in PDAC patients when combining anakinra with gemcitabine, nab-paclitaxel, and cisplatin (AGAP) (81). Although older reports indicated that anakinra alone was not able to induce myeloma cell death, a study involving multiple myeloma patients used anakinra in combination with low-dose weekly dexamethasone, showed an improved survival for over 10 years compared to the controls (82). In breast cancer, the use of pre-clinical mouse models indicated that anakinra treatment decreased tumor growth and bone metastasis (83). Besides, a clinical pilot study investigated the administration of anakinra prior to standard chemotherapy in HER2-negative metastatic breast cancer female patients. The study revealed that 2-weeks of anakinra treatment alone could downregulate the expression of several genes for TLR and IL-1β families, but upregulate the expression of tumor lysis-associated genes like NK and CD8^+^ T-cells (84). These results indicate a promising outlook for the use of anakinra combined with standard chemotherapy in difference cancers. However, the effectiveness of anakinra in antitumor applications needs further investigation through *in vivo* models and later in clinical trials.

Canakinumab is a human anti–IL-1β monoclonal antibody, known for its high specificity to block IL-1β without interference or cross-reactivity with other IL-1 family members. It was approved by the US FDA and European Medicines Agency for treating CAPS (23, 85). Canakinumab has a half-life of a typical IgG1 antibody (124), which gives it an advantage over recombinant IL-1Ra by ensuring the full inhibition of IL-1β over a lengthier period. Interestingly, Canakinumab Anti-inflammatory Thrombosis Outcomes Study (CANTOS), a randomized, double-blinded clinical trial of 10,061 lung cancer and atherosclerosis patients implemented the use of canakinumab, and resulted in a significant reduction of lung cancer-caused mortality. This antitumor effect was evident in lung adenocarcinoma or poorly differentiated large cell cancer due to the few cases of small-cell lung cancers or squamous cell carcinomas (86). Currently, canakinumab is being applied in clinical trials focusing on non-small cell lung cancer (NSCLC), Triple Negative Breast Cancer (TNBC), colorectal cancer and metastatic melanoma. In particular, two ongoing Phase III clinical trials conducted by Novartis pharmaceuticals (CANOPY-1 and CANOPY-2) are currently investigating pembrolizumab plus chemotherapy with or without canakinumab, or docetaxel with canakinumab in NSCLC (ClinicalTrials.gov Identifier: NCT03626545, NCT03631199). The forthcoming results will provide a better insight on in safety and efficacy of using it as combination treatment. However, investigating the use of canakinumab in other cancers remain less prominent, and relatively requires more recognition.

P2X7R mediates NLRP3 inflammasome activation and cytokine release. However, the role of P2X7R in tumor cells is shown to be either pro-tumorigenic or anti-tumorigenic [reviewed in Savio et al. (87)]. Nevertheless, several reports have evaluated the potential of P2X7R antagonists in different cancers and suggested their efficacy in altering tumor cells and suppressing cancer progression. For instance, inhibition of P2X7R caused attenuated tumor proliferation and invasion in PDAC (88), and decreased invasiveness of A253 cells derived from epidermoid carcinoma (46).

Thalidomide, a sedative or hypnotic drug, was used particularly for morning sickness in pregnant women (100). However, it was shown to have an anti-tumor activity due to its antiangiogenic properties (125, 126), and later be an inhibitor of caspase-1 (127). It has been approved as a first-line therapeutic option in patients with advanced multiple myeloma in combination with other chemotherapy drugs because of its anti-tumor activities, resulting in improved response (101, 102). In prostate cancer, the administration of thalidomide alone or in combination with docetaxel resulted in improved response and overall median survival (103, 104). However, its application in other cancer types, such as metastatic melanoma, NSCLC and hepatocellular carcinoma (105–107), did not show significant usefulness.

In addition, VX-765 (108), Pralnacasan (109), and Ac-YVAD-CHO (110, 111) are other caspase-1 inhibitors which have shown few but promising results in their potential in NLRP3-related diseases. However, their potential as therapeutic targets in cancer was not investigated.

Other compounds include Andrographolide (89, 90) and Parthenolide (96), which mainly target NF-κB signaling pathway, but the later was also shown to directly inhibit NLRP3 inflammasome by interfering with its ATPase activity (128), have also shown promising results in several cancers (129). For instance, andrographolide was shown to suppress cancer cell proliferation, promote apoptosis in colon cancer (91), breast cancer (92, 93), multiple myeloma (94), and enhance the antitumor effect of 5-FU in colorectal cancer (95). Besides, parthenolide have shown positive results in inhibiting tumor cell proliferation in gastric cancer (97), pancreatic adenocarcinoma (98), colorectal cancer (99). However, these two compounds have not been taken further beyond pre-clinical studies.

A number of small-molecule compounds were proposed to show specific inhibitory effects on NLRP3 activation [reviewed further in detail elsewhere (130, 131)]. One example is MCC950, which prevents NLRP3-induced ASC oligomerization, leading to the inhibition of both canonical and non-canonical NLRP3 inflammasome activation as well as IL-1β secretion, presenting it as a promising agent in NLRP3-related diseases (112). Mechanistic studies have revealed that MCC950 directly binds to the Walker B motif of the NLRP3 central NACHT domain, blocking the hydrolysis of ATP and thus the formation of NLRP3 inflammasome. This action is independent of K^+^ efflux, Ca2^+^ flux, or NLRP3–ASC interactions, and occurs without interfering with TLR signaling or the priming step of NLRP3 activation (112, 113, 130). The use of MCC950 in head and neck squamous cell carcinoma was shown to delay tumorigenesis and improve the antitumor response by reducing the numbers of MDSCs; regulatory T cells (Tregs) and TAMs (48). Besides, MCC950 treatment in MDS was sufficient to halt restore effective hematopoiesis by inhibition of pyroptosis (55). Furthermore, we have recently reported that the use of MCC950 in *Kras*^*G*12*D*^-mutant leukemia mouse models improves myeloproliferation and cytopenia phenotypes, by attenuating NLRP3 inflammasome (49). However, despite its promising potential in Parkinson's disease (132), preclinical and clinical reports studying MCC950 in cancer remain rather limited.

Oridonin is a major bioactive component of herbal plant *Rabdosia rubescens*, and is widely used as an over-the-counter (OTC) herbal medicine for the treatment of inflammatory diseases (130). Studies have shown that Oridonin can specifically inhibit NLRP3 inflammasome activation, where NACHT domain and Oridonin share cysteine 279 binding site (114). The ability of Oridonin to suppress cell proliferation was previously demonstrated in breast (133) ovarian (115) and esophageal (116) cancers. On the other hand, CY-09 (117), OLT1177(118, 119), Tranilast (120, 121) present as promising specific NLRP3 inhibitors. However, their potential in NLRP3-related cancers has not been investigated yet.

In conclusion, despite the promising prospective of the compounds mentioned above, further studies are still needed to fully understand their therapeutic potential in NLRP3-related diseases, especially in cancers.

## Summary

Despite the well-characterized crucial functions for NLRP3 inflammasome in the immune system, their roles in cancer remain rather complicated and elusive. The double-edged sword effect of NLRP3 inflammasome in cancer appears to be dependent on several factors, including its levels of expression, downstream effector molecules (i.e., IL-1β or IL-18), cancer type, stages of tumorigenesis as well as the potential presence of mutations affecting NLRP3 expression. Therefore, in order to further understand these roles, future research needs to address several points: (i) driving factors of NLRP3 inflammasome activation in tumors, such as oncogenic mutations or mutations of inflammasome components, (ii) possible cross-talk pathways and molecules interacting and affecting the regulation of NLRP3 inflammasome, (iii) effects of TME and its components on NLRP3 inflammasome activation and vice versa, (iv) effect of NLRP3 inflammasome on the regulation of immune cells, antitumor immunity and efficiency of immunotherapy. In summary, targeting the NLRP3 inflammasome or its downstream pathways, either solely or in combination with chemotherapy or other immunotherapeutic approaches, hold a promising potential in cancers.

## Author Contributions

SH and RZ performed literature research together, discussed the articles, and wrote the manuscript together. All authors contributed to the article and approved the submitted version.

## Conflict of Interest

The authors declare that the research was conducted in the absence of any commercial or financial relationships that could be construed as a potential conflict of interest.
